# Integrated Analysis of Proteomic Marker Databases and Studies Associated with Aging Processes and Age-Dependent Conditions: Optimization Proposals for Biomedical Research

**DOI:** 10.3390/proteomes13040057

**Published:** 2025-11-06

**Authors:** Mikhail S. Arbatskiy, Dmitriy E. Balandin, Alexey V. Churov

**Affiliations:** Russian Clinical Research Center of Gerontology, Pirogov Russian National Research Medical University, Ministry of Healthcare of the Russian Federation, 129226 Moscow, Russia

**Keywords:** proteomic biomedical research, proteome, proteomic aging biomarkers

## Abstract

**Background**: The search for reliable aging biomarkers using proteomic databases and large-scale proteomic studies presents a significant challenge in biogerontology. Existing proteomic databases and studies contain valuable information; however, there is inconsistency in approaches to biomarker selection and data integration. This creates barriers to translating existing knowledge into clinical practice and use in biomedical research. This work analyzed experimental proteomic studies, the content of proteomic databases, and proposed recommendations for optimization and improvement of proteomic database formation and enrichment. **Methods**: The study utilized publications devoted to proteomic data acquisition methods, proteomic databases, and experimental studies. **Results**: Methods for obtaining proteomic data were analyzed (Protein Pathway Array (PPA), Tissue Microarray (TMA), Luminex (Bead Array), MSD (Meso Scale Discovery), Simoa (Quanterix), SOMAscan (SomaLogic), Olink (PEA), Alamar NULISA (PEA+), and Oxford Nanopore. A total of 16 proteomic databases were investigated (HAGR, KEGG, STRING, Aging Atlas, HALL, Human Protein Atlas, UniProt, AgeAnnoMO, AgeFactDB, AgingBank, iProX, jMorp, jPOSTrepo, MassIVE, MetaboAge DB, PRIDE Archive). Additionally, 22 proteomic studies devoted to aging and age-associated diseases were analyzed. **Conclusions**: Proteomic databases and experimental studies individually contain valuable information about aging biomarkers. Using data from different sources within biomedical research poses challenges for improving and optimizing methodological solutions for publication selection, database formation, and marker development.

## 1. Introduction

Population aging worldwide has created an acute need for reliable biomarkers that can assess biological age and predict the risk of age-related diseases.

To obtain information about age-related changes in humans, omics studies (genomic, transcriptomic) are conducted, among which proteome studies occupy a special place, since in the DNA–RNA–protein chain, protein is at the end, and it is proteins that determine the functional capabilities of cells [1].

For studying aging processes and age-associated diseases, cohorts with specified parameters are formed. Cohorts are formed with various designs: broad age range—LonGenity, INTERVAL (Lehailler et al., 2019) [2]—18–95 years, CN-Beijing-2012 (Lu et al., 2012) [3]—18–82 years; InCHIANTI study (Tanaka et al., 2020) [4]—21–102 years; narrower age range—LonGenity (Sathyan et al., 2020) [5]—65–95 years, PIVUS (Lind et al., 2019) [6]—70–80 years; cross-sectional studies—LonGenity, INTERVAL (Oh et al., 2023) [7]; longitudinal studies—CHS, FOS (Liu et al., 2022) [8], different numbers of participants—IT-2020 (Santos-Lozano et al., 2020)—18 participants [9], LonGenity (Sathyan et al., 2020) [5]—1025 participants. Cohorts formed within studies can be combined into meta-analyses containing a larger number of cohorts—for example, in the study by Coenen et al., 2023 [10], cohorts ABF300, HERITAGE, LonGenity, and deCODE were combined. At this stage, researchers face the problem of data heterogeneity, as obtaining data from participants of each cohort requires different methods for obtaining proteoforms with varying sensitivity. This leads to the problem of non-overlapping results from individual studies. This occurs due to the absence of data preprocessing and normalization before combining them into a unified array for analysis and comparison. These problems are described in the Section 3 of this article.

Proteomic databases aim to combine data from various cohorts and studies. When analyzing 16 databases dedicated to studying aging and age-associated diseases or containing such a section, several observations were made. Only two databases contain statistical information about the methods used to obtain proteomic data. This is extremely important when conducting large-scale biomedical research to account for the sensitivity of each method at the data normalization stage [11,12,13]. The problem of lack of database maintenance is common, so in our study this fact is considered but not discussed. Access to databases is an important characteristic as it allows automation of the database querying process. Not all databases have programmatic access (API) capabilities. From most databases, data can only be obtained through downloading. There is no comprehensive description of the information contained in the databases, which complicates the effective use of such data. The listed problems are examined using specific databases as examples in the Section 3.

## 2. Materials and Methods

### 2.1. Preliminary Document Processing

To conduct a comprehensive analysis of proteomic databases and experimental aging studies, 74 articles were selected containing information about proteomic databases and large-scale experimental studies of aging processes that yielded proteomic data. Publications containing at least one of the following keywords were used: “proteom”, “aging”, “ageing”, “senescence”, “biomarker”.

### 2.2. Analysis of Proteomic Databases and Resources Dedicated to Aging Research

For the analysis, specific proteomic databases dedicated to aging research were used, as well as proteomic databases that contain a section devoted to aging and repositories containing a large number of studies focused on aging research. Each database website was analyzed according to several criteria: accessibility for research, updates, curation, presence of a search system, availability of statistics, presence of programmatic access (API) or data download capabilities.

### 2.3. Analysis of Experimental Proteomic Studies

For the analysis of experimental studies, works containing results of proteomic analysis dedicated to studying the aging process and age-associated diseases were selected. In cases where the work indicated the cohorts used, the list of selected studies included original works devoted to the formation of such cohorts.

### 2.4. Generation of Venn Diagrams and UpSets

To obtain the final table, sequential integration of data from multiple proteomic studies was performed. First, all proteins were extracted from all studies and databases, and their identifiers were standardized to a unified UniProt format, removing duplicates and synonyms. Then a cross-reference matrix was constructed, where for each protein the frequency of its detection in different studies was calculated. Using the obtained matrices, intersection histograms and Venn diagrams were constructed using Python 3.13.9 libraries upsetplot==0.9.0 and venn==0.1.3, respectively.

## 3. Results

### 3.1. Methods of Obtaining Proteomic Data

Modern proteomics offers a broad arsenal of methodological approaches, each with unique advantages and limitations for aging biomarker research. Our analysis identified ten main technological approaches that shape the contemporary landscape of aging proteomics research and critically influence the quality and reproducibility of the data obtained [14,15,16].

Liquid chromatography–tandem mass spectrometry has established itself as the gold standard for deep proteome analysis, providing simultaneous identification and quantitative measurement of thousands of proteins. This method combines the advantages of liquid chromatography for effective separation of proteins with the high accuracy of tandem mass spectrometry for identification and quantification. Analyses of key aging cohorts show that LC-MS/MS accounts for roughly 45% of high-quality proteomic datasets in aging databases, demonstrating deep coverage with an average of 2500–4000 proteins per study compared with other methodologies [17,18].

Targeted mass spectrometry provides precise quantitative definition of pre-selected panels of proteins, ensuring high reproducibility and clinical applicability.

The SOMAscan technology presents an innovative approach to large-scale protein measurement, using DNAaptamers for capture and quantitative determination of proteins. This platform has shown strong performance in aging studies, with simultaneous measurement of 1301 to 4979 proteins depending on platform version. Cross-cohort analyses identified consistent SOMAscan signatures of aging, including a reliable panel of 76 proteins achieving a correlation of 0.94 with chronological age across diverse populations.

The Proximity Extension Assay (PEA) method enables multiplexed immunoassays with high specificity and a wide dynamic range. The Human Disease Blood Atlas used this technology to analyze 1162 genes in more than 6000 patients with 59 diseases, providing crucial data for cross-bridging disease and aging [19,20].

Protein microarrays offer a cost-effective, targeted analysis of specific protein sets using immobilized antibodies. Although these systems have lower throughput compared with mass spectrometry, microarrays allow focused analysis of predefined biomarker panels with clinical accuracy [21].

Thus, the modern methodological arsenal of proteomics represents a complementary spectrum of technologies, each occupying a specific niche in aging research. LC-MS/MS dominates as the preferred method for deep proteome profiling with coverage of thousands of proteins, while targeted mass spectrometry provides precise validation of biomarkers [22,23,24,25]. High-throughput platforms such as SOMAscan and PEA demonstrate excellent capability for simultaneous analysis of hundreds to thousands of proteins with high reproducibility across cohorts, which is crucial for detecting conserved aging signatures [26]. Protein microarrays, despite limited throughput, retain clinical relevance for targeted analysis of predefined biomarker panels. The choice of optimal methodology should be based on balancing depth of analysis, reproducibility, cost-effectiveness, and the study’s ultimate goals, to maximize the informativeness and clinical applicability of obtained aging proteome biomarker data (Supplementary Materials: Tables.docx (Tables S1 and S2)).

### 3.2. Representation of Proteomics Data Acquisition Methods in Databases

Statistical information about the representation of proteomics data acquisition methods is contained in **iProX**. Out of 5791 studies, the following distribution by proteomics data acquisition methods is observed: Q Exactive (1077); Q Exactive HF (786); timsTOF Pro (690); Q Exactive HF-X (617); Orbitrap Fusion Lumos (458); Orbitrap Fusion (446); Q Exactive Plus (430); Orbitrap Exploris (320); TripleTOF 5600 (236); LTQ (153); others (1047).

The **jMorp** database lists proteomics information acquisition methods for 501 samples: nanoLC-Q-FT-IT/MS, nanoLC-FT-IT/MS, and nanoLC-Q-FT/MS, but does not specify the number of studies for each method.

In other databases, information about how the proteomics data included in the database were obtained is absent. Given that all proteomics data acquisition methods have different sensitivity, the absence of such data may be critical.

### 3.3. A Brief Overview of Main Proteomics Databases and Repositories Containing Information on Aging Processes

Over the past decades, a large number of proteomics databases containing proteome information have been created. Some of them contain information about aging processes and age-associated diseases [9,27,28,29]. The **HAGR** (Human Ageing Genomic Resources) database includes six main databases. **GenAge** focuses on aging-related genes and contains 307 genes associated with human aging. In **KEGG** (Kyoto Encyclopedia of Genes and Genomes), the Pathways section was selected, which presents metabolic pathways and signaling pathways, as well as the Diseases section, which describes genetic information related to various diseases and their molecular mechanisms, namely Alzheimer’s disease (hsa05010), Parkinson’s disease (hsa05012), Huntington’s disease (hsa05016), Type 2 Diabetes Mellitus (hsa04930), Cardiovascular diseases (hsa05410, hsa05412), Osteoporosis (hsa05022), and Cancer Pathways (hsa05200). In STRING, the following aging-related processes were selected: GO:0007568 (Aging, GO Process)—171 proteins; GO:0090398 (Cellular senescence, GO process)—60 proteins; GO:0090399 (Replicative senescence), GO Process—16 proteins; MAP-2559583 (Cellular senescence, Reactome)—163 proteins, and map04218 (Cellular senescence, KEGG)—150 proteins. The **Aging Atlas** database is the only one that presents the classification of 503 genes according to various hallmarks of aging [30]. On the **HALL** (Human Aging and Longevity Landscape) platform, the “Proteomics module” section contains data from 28 studies and has compiled an extensive collection of 200 age-changing proteins from 16 cohorts including 53,164 people. The age of participants in these studies ranges from 16 to 105 years. The **Human Protein Atlas** database consists of eight separate resources, each dedicated to a specific aspect of human protein analysis. Of 1162 proteins, only 551 are associated with a specific disease or characterized as Disease variant (Aortic aneurysm (4); Cancer-related genes (283); Cardiomyopathy (6); Intellectual disability (9); and Neurodegeneration (29)). The **UniProt** database contains information on 5175 proteins associated with various diseases, including age-related ones. These data were derived from the following resources available in the public domain: [Uniprot, https://www.uniprot.org/diseases?query=* (accessed on 25 July 2025)]. From all diseases presented in the database, only age-associated diseases were selected. There were 24 such diseases [31,32] (Table 1). For each of these diseases, lists of proteins represented in the database were obtained. In total, 411 proteins were identified, which are presented in the Supplementary Materials.

**AgeAnnoMO** represents a multi-omics annotation knowledge base for animal aging research, including 136 datasets from eight modalities covering 8586 samples from 50 species with comprehensive annotation of genes, proteins, metabolites, mitochondrial genes, microbiota, and age-specific TCR and BCR sequences associated with aging traits. From age-associated, age-specific TCRBCR, 77,694 receptors were found; from Differential gene expression_scRNA, 1485 proteins from testis, 1517 from cerebrospinal fluid, and 1276 proteins from blood were found. From Age-correlated protein, 664 in plasma, 81 serum, and 857 skeletal muscle proteome were found. From Differential protein, 3555 in plasma and 2446 proteins in skeletal muscle proteome were found [33]. The **AgeFactDB** database contains 862 aging-associated factors. Of these, 572 are putative (Computational Analysis) and 290 are yes (Experimental Analysis) [34]. In **AgingBank**, the human_experimentally supported associations section contains 1133 factors. Of these, there are 4 alternative splicing, 3 circRNA, 6 CNV, 7 enhancer, 307 gene, 2 gene silence, 40 histone modification, 16 lncRNA, 187 methylation, 107 miRNA, 1 RNA editing, 244 SNP, 109 somatic mutation, and 32 TF [35]. The **jMorp** database contains 501 plasma samples obtained using nanoLC-Q-FT-IT/MS, nanoLC-FT-IT/MS, and nanoLC-Q-FT/MS methods. There is also information on 270 peptides [36].

The **iProX** repository contains 2521 datasets for humans [37]. **jPOSTrepo** (Japan ProteOme STandard Repository) is a Japanese proteomics data repository for rapid sharing of mass spectrometry data through a web browser, with the capability to store data prior to article publication and assign unique identifiers [38]. **MassIVE** (Mass spectrometry interactive virtual environment) contains Public Datasets: 17,711; Proteins: 191,740; Number of Files: 11,158,091; and Peptides: 9,906,636 [39]. **MetaboAge** is not a proteomics database but contains information about metabolome changes during aging, including signaling pathways integrated with KEGG [40]. **PRIDE Archive** is a proteomics research database with the ability to search by diseases (among age-related conditions, only Alzheimer’s disease is available) [41]. Information about the main content of the databases can be seen in Table 2 (Supplementary Materials: Tables_eng.pdf (Tables S3 and S4); Figure S1).

**Figure 1:**
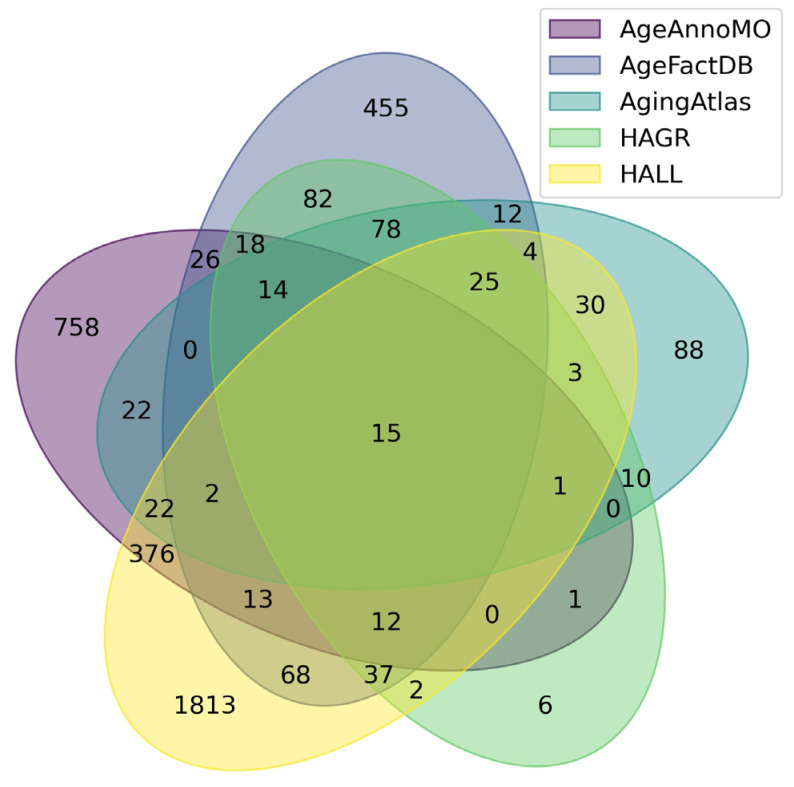
Venn diagram showing intersections of results from five databases for studying plasma/serum proteins.

**Figure 2:**
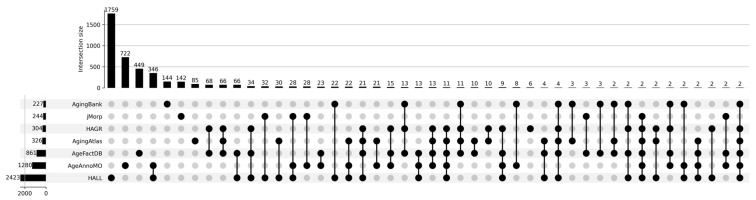
UpSet plot showing intersections of results for databases AgingBank, jMorp, HAGR, AgingAtlas, AgeFactDB, AgeAnnoMO, and HALL.

Figure 1 shows a Venn diagram for five major databases. It can be seen that the intersection between the five databases comprises only 15 proteoforms, which indicates strong inconsistency in results when studying the aging process. The small overlap between aging marker databases is explained by a combination of technical and biological factors. Technical differences include the use of different analytical platforms and sample preparation methods, which affects detection sensitivity and the spectrum of detected molecules. Differences in study design, participant selection criteria, and age group definitions create methodological heterogeneity. Biologically, this is due to the tissue specificity of aging processes, as each tissue has unique metabolic characteristics and gene expression patterns. Inter-individual variability associated with genetic differences, lifestyle, environment, and comorbidities leads to the identification of population-specific markers. The multiplicity of aging mechanisms means that different studies may focus on different aspects of this process—mitochondrial dysfunction, inflammation, proteostasis disruption, or genomic instability. Lists of proteoforms are presented in the Supplementary Materials up-set_analysis_results_DB. Figure 2 shows that the most significant intersections are 346 proteoforms between two databases, AgeAnnoMO and HALL; 66 proteoforms between three databases, AgeFactDB, AgingAtlas, and HAGR; 34 proteoforms between three databases, HAGR, AgeFactDB, and HALL; and 21 proteoforms between four databases, HAGR, AgingAtlas, AgeFactDB, and HALL.

### 3.4. Analysis of Selection Criteria for Experimental Studies for Addition to Database Bibliographies

Modern biomedical databases represent a complex ecosystem where each platform develops unique publication selection criteria to ensure high quality and relevance of the presented information. The conducted analysis of 16 databases revealed a multilayered system of requirements, reflecting the evolution of scientific standards and the specificity of various research areas.

Temporal selection criteria demonstrate significant variability depending on the type and purpose of the database. Fundamental repositories, such as UniProt and RefSeq, do not establish temporal limitations, recognizing the value of historical data for understanding biological processes [42]. In contrast, specialized aging and proteomics databases, including Aging Atlas, MINDMAP, and GISAO.db, focus on publications from the last decade, reflecting the dynamic development of modern high-throughput technologies. An intermediate position is occupied by mass spectrometry databases PRIDE and iProX, which began systematic data collection from the moment of proteomics data format standardization.

Journal quality requirements, measured by impact factor, form a clear hierarchy of scientific significance. The most stringent criteria (IF ≥ 4.0–5.0) are applied by clinically oriented databases HAGR, AgeFactDB, and CPTAC, which are conditioned by the need to ensure high data reliability for translational research. A medium level of requirements (IF 2.0–3.5) is characteristic of databases Aging Atlas, MINDMAP, and RefSeq, balancing between quality and data volume. The most flexible criteria (IF ≥ 1.0) are applied by international repositories PRIDE, iProX, and regional databases such as jPOSTrepo, which promote broader coverage of scientific results [43,44,45] (Supplementary Materials: Tables_eng.pdf (Table S4)).

### 3.5. Methodological Foundations for Biomarker Selection and Heterogeneous Data Integration

The development of reliable aging biomarker panels requires complex and robust methodological approaches for integrating heterogeneous data sources [46,47].

The HAGR database (GenAge) uses an eight-criteria framework that has significantly evolved since 2004. The current methodology includes direct evidence linking genes to human aging, evidence from mammalian model organisms, associations with human longevity and age-related phenotypes, evidence from non-mammalian models, cellular system evidence, regulatory or control relationships with aging genes, pathway or mechanism connections, and downstream effects of aging-related pathways. However, this approach suffers from fundamental limitations, including the absence of representation level considerations and lack of quantitative weighting systems [48].

The HALL database implements the most stringent selection methodology, requiring compliance with the American Federation for Aging Research (AFAR) criteria for aging biomarkers. The database uses manual curation from published studies with three different biomarker categories: aging-associated biomarkers, longevity and healthy aging biomarkers, and biomarkers for 52 age-associated diseases. This approach ensures high clinical relevance but introduces notable selection bias toward well-studied proteins [49,50].

The Human Protein Atlas uses experimental validation requirements with specific algorithmic approaches for publication analysis, representing the most stringent technical validation standards. However, the database lacks aging-specific selection criteria, limiting its utility for targeted aging biomarker development.

Our analysis identified five fundamental gaps in existing biomarker selection approaches.

First, the absence of quantitative representation thresholds means that only 2 of 7 analyzed databases include actual protein representation levels in selection criteria despite the fact that representation magnitude is critical for clinical utility of biomarkers.

Second, lack of demographic stratification means that current methodologies do not account for age, sex, ethnicity, and comorbidity factors that significantly influence protein representation patterns and biomarker validity.

Third, insufficient cross-platform validation requirements mean that existing selection criteria do not mandate validation across multiple analytical platforms, leading to method-dependent biomarker candidates with limited transferability.

Fourth, population-specific validation gaps mean that geographical and ethnic diversity considerations are absent in most selection frameworks, limiting the global applicability of identified biomarkers.

Fifth, temporal stability assessment means that long-term stability evaluations and intra-individual variability are not systematically included in selection criteria.

### 3.6. Analysis of Experimental Proteomic Study Designs

For the analysis, cohorts ABF300 (Arthur et al., 2021) [51], HERITAGE (Robbins et al., 2021) [52], LonGenity (Sathyan et al., 2020) [5], deCODE (Ferkingstad et al., 2021) [36], New England Centenarian Study (Sebastiani et al., 2021) [53], and COVIDome (Sullivan et al., 2021) [54] were used. Interestingly, these six studies had already been analyzed in another study (Coenen et al., 2023) [10]. The cohorts Covance (Williams et al., 2019) [55], LonGenity (Sathyan et al., 2020) [5], Stanford Alzheimer’s Disease Research Center (Wilson et al., 2022) [56] and Knight Alzheimer’s Disease Research Center (Berg et al., 1998) [57] were described in the study Oh et al., 2023 [7]. Two separate studies were (Xu et al., 2020) [58] and Sicilian plasma cohort (Siino et al., 2022) [59]. Information from the study (Tanaka et al., 2020) [4] was also used, which utilized data from the InCHIANTI study (Zuliani et al., 2017) [60]. Two long-standing studies, the Cardiovascular Health Study (CHS) (Fried et al., 1991) [61] and Framingham Heart Study (FHS) (Feinleib et al., 1975) [62], were analyzed in the work (Liu et al., 2022) [8].

#### 3.6.1. The Following Studies Were Also Used for the Research

1. In the work by Tanaka et al., 2018, a proteomic profile of aging in healthy individuals was identified [63]. Plasma samples measured using the SOMAscan method were used from 240 healthy men and women aged 22 to 93 years.

2. Lehallier et al., 2019 identified changes in the human plasma proteome throughout life to better understand the biological processes of aging and identify potential targets for treating age-related diseases [2,64]. The study included 4263 people aged 18 to 95 years.

3. In the work by Sathyan et al., 2020, the plasma proteomic profile associated with age, lifespan, and all-cause mortality in elderly individuals was studied using the SomaScan platform with 1025 people from the LonGenity cohort aged 65–95 years, of whom 55.7% were women [5]. This was a study of age-related changes in blood plasma proteins among a healthy Sicilian cohort, including centenarians.

4. The study by Siino et al., 2022, aimed to identify unique protein patterns associated with healthy aging and longevity [59]. The study included 86 participants aged 22 to 111 years. A total of 410 proteins were identified and quantitatively assessed.

5. The goal of the study by Lu et al., 2012 was to identify aging biomarkers in Chinese Han adults through blood plasma proteomic analysis [3]. This study aimed to understand changes in age-related protein concentrations and their potential use as biological markers of aging. The total number of study participants was 1890 people, of whom 1136 were men and 754 were women. Participant ages ranged from 18 to 82 years.

6. The study by Ye et al., 2019, focused on identifying proteomic characteristics associated with longevity and understanding the molecular mechanisms that may contribute to long life [65]. A total of 66 plasma donors participated in the study. Of these, 33 people were descendants of centenarian families (longevity group), and the remaining 33 were a control group from families without a history of longevity. The study identified 525 quantitatively determined plasma proteins, of which 12 were found to be differentially represented between the two groups.

7. The goal of the study by Santos-Lozano et al., 2020, was to conduct a comparative analysis of the plasma proteome in healthy centenarians (*n* = 9, 5 women, age 100–103 years) with preserved mobility (paradigm of “successful” aging) and controls who died from major age-related diseases before reaching expected lifespan (*n* = 9, 5 women, age 67–81 years) with impaired mobility (paradigm of “unsuccessful” aging) [9]. 18 people participated in the study, divided into two groups: 9 healthy people aged 100 years and 9 control participants. It was found that the abundance of 49 proteins and 86 pathways differed between the two groups.

8. The goal of the study by Xu et al., 2020 was to identify differentially represented proteins specific to different age groups and systematically characterize changes in blood plasma protein composition to propose potential biomarkers for the diagnosis and treatment of age-related diseases [58]. The study included 118 healthy adult participants who were divided into three age groups: 21–30 years (young), 41–50 years (middle age), and ≥60 years (elderly).

9. The study by Lind et al., 2019, aimed to investigate changes in blood plasma protein levels in elderly people with age, as well as their relationship with kidney function and hemoglobin levels [6]. A total of 1016 people participated in the study, all of whom were 70 years old at the start of the study. Measurements were conducted at ages 70, 75, and 80 years. A total of 84 proteins were investigated, of which 61 proteins showed significant changes over 10 years, with most of them increasing.

10. The goal of the study by Tanaka et al., 2020, was to identify age-related biomarkers in blood plasma that could predict human lifespan [4]. A total of 997 people aged 21 to 102 years participated in the study. A total of 651 age-related proteins were identified (506 were elevated, 145 decreased with age).

11. The study by Wang et al., 2020, aimed to identify plasma proteins associated with longevity using proteomic analysis methods [39]. The study was conducted with participants from the Bama longevity group and a control group, although the exact number of participants was not specified. A total of 175 differentially abundant proteins (DEPs) were identified, which are mainly associated with complement and coagulation cascades, carbohydrate and lipid metabolism, and actin cytoskeleton regulation.

12. The study by Coenen et al., 2023, aimed to identify proteins in human blood plasma associated with aging [10]. Data from four independent large datasets using the SomaScan platform were integrated. The study included four different cohorts: Arthur et al. [51], with 150 participants; Robbins et al., with 745 participants; Sathyan et al. [5], with 1025 participants; and Ferkingstad et al. [36], with 35,559 participants. 273 plasma proteins that were significantly associated with aging were identified.

13. The study by Liu et al., 2022, aimed to determine the proteomic signature in blood plasma associated with changes in physical function, measured by decreased walking speed and grip strength in middle-aged and elderly people [8]. This may help understand the biological mechanisms of physical aging and identify new biomarkers for preventing physical function decline. The study included 2854 people from the Cardiovascular Health Study (CHS) and 1130 participants from the Framingham Offspring Study (FOS), aged 29 to 100 years.

14. The goal of the study by Oh et al., 2023, was to assess how organ aging manifests differently in people and how this may affect age-related diseases [7]. A total of 5676 adults from five independent cohorts covering a wide age range participated in the study. The study showed that about 20% of participants experience accelerated aging in one organ, and 1.7% have accelerated aging in multiple organs, which is associated with 20 to 50% higher mortality risk.

#### 3.6.2. Additionally, Eight Meta-Analyses Were Analyzed

1. In the study by Salignon et al., 2023, levels of proteins and small RNAs in 103 human blood plasma samples were studied [66]. First, a two-step mass spectrometry approach was used to measure 612 proteins to select and quantitatively determine 21 proteins that changed in quantity with age. Proteins increasing with age were enriched with complement system components. Next, small RNA sequencing was used to select and quantitatively determine a set of 315 small RNAs that changed in quantity with age. Most of these were microRNAs (miRNAs) whose expression levels decreased with age and which are predicted to target genes associated with growth, cancer, and aging. Blood plasma from a cohort of 103 North Americans aged 20 to 83 years, with an average age of 55 years, was used to study age-related molecular changes in humans. Measurements were conducted only in a subgroup from the cohort, which included 19 young people (aged 20 to 30 years) and 25 elderly people (aged 65 to 76 years).

2. The LiP-MS method allowed reproducible identification of structural changes caused by alterations in protein folding, assembly state, interaction with other molecules, or post-translational modifications. Comparative analysis of protein structural states between young and old cells revealed age-dependent structural differences in 468 proteins, which included 1272 conformationally specific peptides (total proteins detected: 2833) (Jurgita Paukštytė et al., 2023) [10].

3. The goal of the study by Oh et al., 2023, is to identify a set of proteins in human plasma associated with aging by integrating data from four independent, large-scale datasets using the SomaScan platform [7]. Using this approach, a set of 273 plasma proteins significantly associated with aging (aging proteins, AP) was identified in these cohorts consisting of healthy individuals and people with comorbidities, and their biological functions were highlighted.

4. In the study by Coenen et al., 2023, measurements of 4979 proteins in 5676 subjects across five independent cohorts were conducted to create an organ-specific plasma proteome map and organ aging models [10]. Organ-specific proteins were determined, which were used to train aging models using the LASSO method for 11 major organs.

5. In the work by Argentieri et al., 2024, a method for assessing “proteomic age” was developed in the UK Biobank database (*n* = 45,441) using a proteomic platform consisting of 2897 plasma proteins, and the possibility of using it to predict major diseases and mortality in different populations was investigated [67,68]. A total of 204 proteins were identified that accurately predict chronological age (Pearson correlation coefficient r = 0.94), and it was found that proteomic aging is associated with the occurrence of 18 major chronic diseases (including heart, liver, kidney and lung diseases, diabetes, neurodegeneration and cancer), as well as with polymorbidity and all-cause mortality risk.

6. In the work by Moaddel et al., 2021, 232 proteins were identified whose plasma concentration was significantly associated with age in one direction in at least two different studies and which are associated with age in at least one other tissue (besides blood), regardless of direction [63]. Results for proteins meeting these criteria were classified into 4 categories.

7. The systematic review by Johnson et al., 2020, includes 36 different proteomic analyses, each of which identified proteins that significantly change with age [69]. A total of 1128 proteins were found that were recorded in at least two or more analyses, and 32 proteins that were recorded in five or more analyses.

8. A total of 12 studies with mice, 11 with rats, and 4 with humans were analyzed, comprising nearly 5 million gene expression measurements from more than 400 individual samples. A meta-analysis of gene expression profiles associated with aging was conducted using 27 datasets obtained from mice, rats, and humans. The results reveal several common features of aging, including 56 genes that are consistently highly expressed with age, of which APOD was the most significant, and 17 genes that are underexpressed with age (Magalhães et al., 2011) [70].

Basic information about the experiments used in the study can be found in Table 3. (Supplementary Materials: Tables_eng.pdf (Table S5); Figure S2).

**Figure 3:**
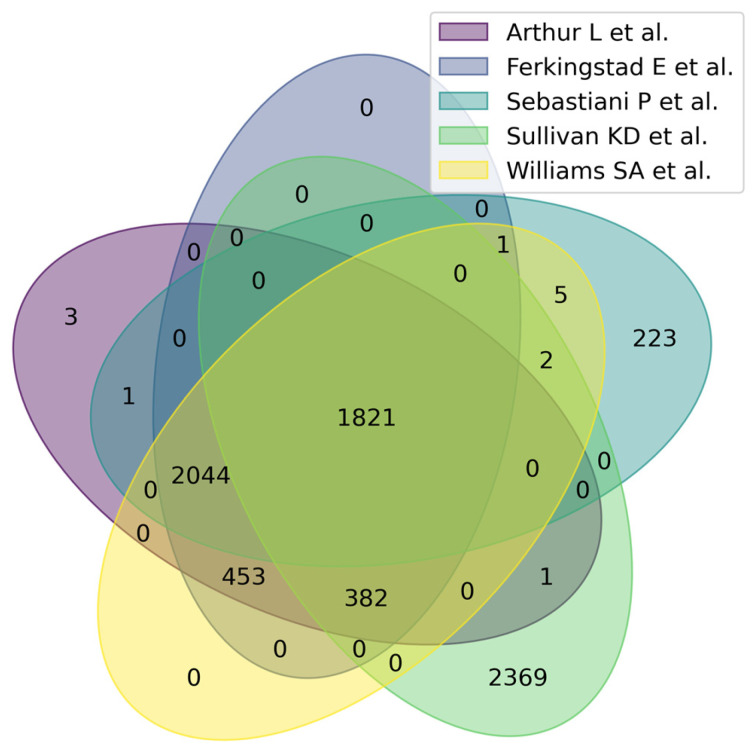
Venn diagram showing intersections of results from five independent studies on plasma/serum proteins.

**Figure 4:**
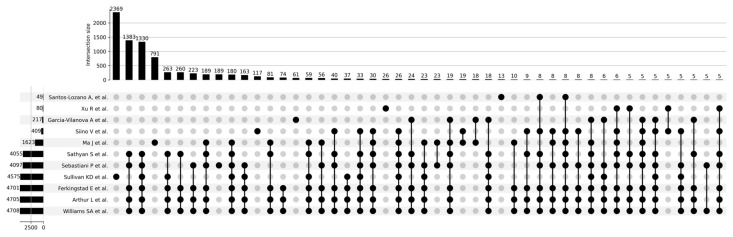
UpSet plot showing intersections of results for Santos-Lozano et al. [9], 2020; Xu et al., 2020 [58]; Garcia-Valanova et al. [17]; Siino et al. [59], 2022; Ma et al. [37]; Sathyan et al., 2020; Sebastiani et al.; Sullivan et al. [54]; Ferkingstad et al. [36]; Arthur et al. [51]; Williams et al. [55].

Figure 3 shows a Venn diagram for five main studies. It can be seen that the intersection between the five studies comprises 1821 proteoforms, which indicates good consistency of results when studying the aging process. Figure 4 shows that the most significant intersections are 180 proteoforms between 7 studies (Ma et al. [37], Sathyan et al., 2020 [5], Sebastiani et al. [53], Sullivan et al. [54], Ferkingstad et al. [36], Arthur et al. [51], Williams et al. [55]) and 26 proteoforms between 8 studies (Siino et al., 2022 [59], Ma et al. [37], Sathyan et al., 2020 [5], Sebastiani et al., Sullivan et al. [54], Ferkingstad et al. [36], Arthur et al. [51], Williams et al. [55]) Lists of proteoforms are presented in the Supplementary Materials upset_analysis_results_Research.

### 3.7. Search for Proteomic Markers Associated with Aging and Age-Related Diseases in Experimental Proteomic Studies

To search for aging biomarkers obtained from studying widespread cohorts, 22 studies dedicated to investigating the aging process and age-associated diseases were analyzed. The search results are presented in (Supplementary Materials: Tables_eng.pdf (Table S6)).

### 3.8. Data Integration Challenges for Heterogeneous Data

The common problem with all the aforementioned resources is that data from different sources and obtained by different methods are stored and published without unified normalization. Each method (LC-MS/MS, Luminex, Simoa, SOMAscan, Olink, protein arrays, etc.) has its own sensitivity, dynamic range, and error specificity. For example, Simoa and SOMAscan platforms demonstrate detection limits at the femto- and attomolar levels, while traditional ELISA or multiplex assays are typically sensitive at the picogram level. Mass spectrometry can identify thousands of proteins but usually falls short of specific immunoplatforms in detecting very low-abundance proteins. The Olink platform claims the ability to measure 5000 proteins with very high sensitivity; however, comparisons of different methods show that correlation between them is far from ideal. For instance, studies note that results from Luminex, MSD, and other systems sometimes differ by orders of magnitude.

Thus, if one database contains protein data obtained by mass spectrometry and another uses a combination of Luminex and SOMAscan, their raw values are not directly comparable even when using absolute concentrations. This is precisely why complex normalization is required for proper comparison and integration of such heterogeneous datasets. Data normalization (standardization of ranges, removal of batch effects) is a critically important step in integrated analysis [66]. For example, Z-transformation or normalization by housekeeping proteins is often used to bring all measurements to a unified scale. However, practically all the mentioned proteomic databases lack such a unified approach. Data are uploaded in their original form from experimental papers, where each author used their own analysis method and metrics. Typically, databases do not recalculate values to a common scale—they simply combine records from different studies. The problem is compounded by the fact that different databases use different protein datasets. Regarding bead array methods and aptamer-based methods, there is also a difference in analyte detection related to the use of different antibody clones or aptamers. The resolution level of proteomics remains low, while transcriptomics has already reached single-cell level with full isoform resolution [71].

Thus, storage of proteomic data by different methods creates a data incompatibility problem. At present, the general conclusion is as follows: it is necessary to either develop unified measurement standards (which is extremely difficult) or conduct special cross-platform calibrations before comparison. Until this is done, it is important to remember that direct comparison of raw data from different sources provides a distorted picture.

## 4. Discussion

In our work, we tried to draw the attention of readers and the scientific community to rarely discussed issues in proteomics. These can be divided into three main parts according to the objects of analysis—databases, experimental studies, and methods for obtaining proteomic data.

### 4.1. Proteomic Databases

Proteomic databases may be dedicated solely to aging research, or they may have a section containing information about genes or proteins that characterize the aging process [72].

Some databases contain information not only about the characteristics of studies from which data were extracted, but also the method of obtaining proteomic data, which is very important for the task of integral analysis of information from different databases, since each method has its own sensitivity and determines the final list of identifiable proteins. In our subsequent research, we plan to analyze the representation of methods for obtaining proteomic data. For this purpose, we will analyze publications whose information was included in the database.

Only a few databases are regularly updated and curated. Many databases have not been maintained for more than 10 years but contain extremely important and useful information.

Not all databases have API connectivity, and information from them can only be obtained by downloading data in csv, tsv, txt, xml formats, etc.

### 4.2. Experimental Studies

Most modern proteomic studies use well-known, widespread cohorts that were formed to study various aspects of aging. In new works, only the research focus changes, while the cohorts remain the same and new ones are not formed.

When using previously formed cohorts in new studies, it is not always possible to find the original work that describes the inclusion and exclusion criteria, age ranges, number of participants, and number of obtained proteoforms. This complicates the task of selecting suitable cohorts for new biomedical research. In our next work, we intend to create a registry of existing cohorts for proteomic studies and structure related studies that use data from these cohorts. Despite the obviousness of such a solution, we were unable to find similar works. This approach will allow using not only the cohorts themselves, but also the results of studies conducted on their material, which will significantly enrich and improve subsequent research.

### 4.3. Methods for Obtaining Proteomic Data

Methods for obtaining proteomic data have a specific purpose in proteomics, related to sensitivity for identifying proteoforms in biological material. In this regard, when comparing research results obtained using different methods, significant discrepancies and lack of overlap may be observed. This issue requires a systematic solution and should be considered when entering new obtained results into databases. A solution could be the use of normalization of proteoform abundance values in samples to enable subsequent comparison of abundance across different studies.

### 4.4. Optimization Proposals for Biomedical Research

For conducting large-scale biomedical studies of aging and age-associated diseases, proteomic data from various sources should be used. For this purpose, it is desirable to consider the following features:

As a source of information about detected proteins, consider not only the study that uses information from a particular cohort, but also the original study itself. This will help avoid inconsistencies in values for the number of participants and abundance of detected proteins.

Consider the method of obtaining proteomic data, as this allows accounting for method sensitivity and performing normalization when integrating multiple studies.

When using databases, it is essential to check parameters such as the availability of statistical data, possibility of programmatic access through API, availability of column descriptions for downloadable tables, selection criteria and requirements for publications and data sources from which information entered into the database is extracted, data normalization before database entry, and the availability of clinical data or the possibility of requesting them for more detailed studies.

It is important to note that our approach has limitations regarding proteoform analysis. Proteomes are characterized by significant complexity, including multiple proteoforms of the same protein arising from alternative splicing, post-translational modifications, and other processes. Our analysis does not provide information about specific proteoforms and their functional significance under the studied conditions.

### 4.5. Limitations of the Study

During the preparation of this review, the authors encountered a number of significant limitations that must be considered when interpreting the results. One of the main problems is the lack of a unified protocol for normalizing proteomic data when entering them into databases. Although proteomic data are obtained by various methods, the absence of a standardized approach to their processing significantly complicates subsequent analysis of information from different sources. Particularly notable is the small overlap of aging markers between specialized databases and databases containing sections devoted to age-associated diseases. This is explained by a combination of technical and biological factors. From the technical side, the use of different analytical platforms and sample preparation methods affects detection sensitivity and the spectrum of detectable molecules. Differences in study design, participant selection criteria, and definitions of age groups create methodological heterogeneity. From a biological standpoint, this is due to the tissue specificity of aging processes, as each tissue has unique metabolic characteristics and gene expression patterns. Inter-individual variability associated with genetic differences, lifestyle, environment, and comorbidities leads to the identification of population-specific markers. The multiplicity of aging mechanisms means that different studies may focus on different aspects of this process—mitochondrial dysfunction, inflammation, proteostasis disruption, or genomic instability. An additional limitation is that many modern proteomic studies use known cohorts without their detailed description in the materials and methods section, which makes it difficult to perform statistical calculations that require consideration of the total cohort size, sizes of individual cohorts, age and sex distribution, and other important parameters.

## 5. Conclusions

In this work, we conducted an analysis of methods for obtaining proteomic data and attempted to formulate the problem of accounting for method sensitivity. We collected and structured data on specialized databases and those containing sections devoted to the study of aging and age-associated diseases and analyzed experimental studies to compile a list of proteomic biomarkers of aging.

The most common techniques for obtaining proteomic data in databases according to our study are SOMAscan and LC-MS/MS. Among the 16 databases, there are specialized ones as well as those containing sections devoted to studying the aging process and repositories that store information about research studies. API is available for 5 databases (KEGG, MetaboAge DB, PRIDE Archive, STRING, Uniprot).

Conducting large-scale biomedical research involves using a large number of information sources, and the absence of an established methodology can lead to false results and incorrect conclusions [73]. In this regard, it is necessary to adhere to a developed protocol or use our experience and formulated recommendations for optimizing this process. This work is one in a series of articles that we plan to devote to the analysis of omics data. In our next work, we plan to conduct a detailed analysis of proteomic databases that, in addition to the existing analysis, will contain information about the representation of proteomic data acquisition methods in databases, as well as a catalog of proteomic studies divided into publications devoted to the formation of individual cohorts and publications in which studies were conducted using existing cohorts. This will enable the most efficient conduct of large-scale biomedical research in the future, without wasting time establishing relationships between primary sources and studies that use them. Such an approach will contribute to the formation of completely new, non-duplicating knowledge.

## Figures and Tables

**Table 1:** Number of proteins in the Uniprot database characterizing age-associated diseases.

Age-Related Diseases	Proteins Number
Abdominal obesity-metabolic syndrome	4
Alzheimer disease	21
Anemia	35
Arthritis (including Juvenile arthritis)	No
Autoimmune disease	24
Cardiomyopathy	64
Coronary artery disease	5
Cataract	54
Chronic obstructive pulmonary disease (COPD)	No
Dementia	No
Diabetes mellitus	8
Hertight	No
Hypertension (Essential hypertension)	1
Macular degeneration	26
Osteoporosis	26
Migraine	5
Multiple sclerosis	4
Nephrolithiasis (Kidney Stones)	13
Obesity	19
Osteoarthritis	10
Osteoporosis (listed again)	5
Parkinson disease	46
Rheumatoid arthritis	22
Type 2 diabetes mellitus	24

**Table 2:** Characteristics of major proteomic databases.

No.	Database	Data Volume (2024)	Methodological Requirements
1	AgeAnnoMO	Samples 8586 Dataset 136	Genetic experiments
2	AgeFactDB	Ageing Factors 16,599 Genes 16,450 Compounds 91 Other Ageing Factors 58	Aging factors
3	Aging Atlas	1133 Aging factors	Aging omics data
4	AgingBank	503 genes across various Hallmarks of aging	Aging genes/proteins
5	HAGR	Human genes: 307	Longevity/gerontology
6	HALL	500+ researches	Healthy aging
7	Human Protein Atlas	Consists of 1162 proteins quantified by Proximity Extension Assay (PEA) and 146 proteins quantified by isotope dilution strategies	Immunohistochemistry, transcriptomics
8	iProX	2521 datasets for humans	Proteomic MS data
9	jMorp	Results of proteome analysis of approximately 500 Japanese plasma samples	Morphological data
10	jPOSTrepo	3573 projects are registered. 2668 are opened. 460 species.	Proteomic data
11	KEGG	Pathway maps 580 Human diseases 2963	Pathway mapping
12	MassIVE	Public Datasets: 17,711, Proteins: 191,740, Number of Files: 11,158,091, Peptides: 9,906,636	Any MS data
13	MetaboAge DB	1500+ metabolites	Aging metabolomics
14	PRIDE Archive	Contains over 42,000 datasets	MS/MS data, standard formats
15	STRING	Homo sapiens has 19,488 proteins with network connections	Protein interactions
16	UniProt	220+ million records	Experimental validation

**Table 3:** Basic characteristics of the cohorts used in the study.

No.	Study Title	Number of Samples	Age Distribution	Method Used	Number of Proteins
1	Plasma proteomic signature of age in healthy humans (2018) [63]	240 healthy men and women	22–93 years	SomaScan	217
2	Undulating changes in human plasma proteome profiles across the lifespan (2019) [2]	4263 humans	18–95 years	LC-MS/MS	373
3	Plasma proteomic profile of age, health span, and all-cause mortality in older adults (2020) [5]	1025 people from the LonGenity cohort 55.7% are women.	65–95 years	SomaScan	754
4	Plasma proteome profiling of healthy individuals across the life span in a Sicilian cohort with long-lived individuals (2022) [59]	86 participants	22–111 years	LC-MS/MS	410
5	Profiling plasma peptides for the identification of potential ageing biomarkers in Chinese Han adults (2012) [3]	1890 people (1136 men and 754 women).	18–82 years	LC-MS/MS	44
6	Plasma proteomic and autoantibody profiles reveal the proteomic characteristics involved in longevity families in Bama, China (2019) [65]	66 people (33 people—descendants of long-lived families (longevity group), and the remaining 33—control group from families without a history of longevity).	34–56 years	LC-MS/MS	525
7	Successful aging insights from proteome analyses of healthy centenarians (2020) [67]	18 people, divided into two groups: 9 healthy centenarians and 9 control participants.	67–81 years	LC-MS/MS	49
8	Age-Dependent Changes in the Plasma Proteome of Healthy Adults (2020) [58]	118 healthy adult participants, divided into 3 groups: 21–30 years old (young), 41–50 years old (middle-aged) and ≥60 years old (elderly).	21–93 years	LC-MS/MS	1069 proteins, of which 845 were quantitatively determined.
9	Longitudinal effects of aging on plasma proteins levels in older adults—associations with kidney function and hemoglobin levels (2019) [6]	1016 humans	70 years old at the start of the study. Measurements were conducted at ages 70, 75, and 80 years.	LC-MS/MS	84 proteins, of which 61 proteins showed significant changes over 10 years.
10	Plasma proteomic biomarker signature of age predicts health and life span (2020) [4]	997 humans	21–102 years	LC-MS/MS	651 age-related proteins were identified (506 were overrepresented, 145 decreased with age)
11	TMT-Based Quantitative Proteomic Analysis Reveals Proteomic Changes Involved in Longevity (2019) [47]	66 people (33 people—descendants of long-lived families (longevity group), and the remaining 33—control group from families without a history of longevity)	34–56 years	LC-MS/MS	175 (54 were up-regulated and 121 were down-accumulated)
12	Markers of aging Unsupervised integrated analyses of the human plasma proteome (2023) [36]	Four different cohorts: Arthur et al., with 150 participants, Robbins et al., with 745 participants, Sathyan et al., with 1025 participants, Ferkingstad et al., with 35,559 participants.	16–95 years	LC-MS/MS	273
13	Plasma proteomic signature of decline in gait speed and grip strength (2022) [8]	2854 people from the Cardiovascular Health Study (CHS) and 1130 participants from the Framingham Offspring Study (FOS)	29–100 years	LC-MS/MS	14
14	Organ aging signatures in the plasma proteome track health and disease (2023) [7]	5676 adults from five independent cohorts (KADRC (train), KADRC (test), Covance, LonGenity, SADRC, SAMS)	19–95 years	LC-MS/MS	580
15	Age prediction from human blood plasma using proteomic and small RNA data a comparative analysis (2023) {16}	103 participants	20–83 years	Hyper Reaction Monitoring mass spectrometry (HRM-MS)	21
16	Global analysis of aging-related protein structural changes uncovers enzyme-polymerization-based control of longevity (2023) [32]	Fractions of young (average age 0.6 divisions) and old (average age 4.2 divisions) budding yeast cells.		Limited proteolysis-mass spectrometry (LiP-MS)	468
17	Markers of aging Unsupervised integrated analyses of the human plasma proteome (2023) [36]	37,479 people from four independent large-scale studies. (150, 25–80; 745, 16–66; 1025, 65–95; 35,559, mean 55).	16–95 years	SomaScan platform	5000
18	Organ aging signatures in the plasma proteome track health and disease (2023) [7]	5676 people in five independent cohorts (Covance *n* = 1029, 19–89; LonGenity *n* = 962, 61–95; SAMS, *n* = 192, 60–88; Stanford-ADRC, n = 409, 36–93; Knight-ADRC AD, *n* = 1677, 27–101).	19–95 years	SomaLogic SomaScan assay	4979
19	Proteomic aging clock predicts mortality and risk of common age-related diseases in diverse populations (2024) [67]	UK Biobank (45441, 39–71 years); China Kadoorie Biobank (3977 CKB, 30–78 years); FinnGen (1990 Finnish, 19–78 years).	19–78 years	PEA	204
20	Proteomics in aging research A roadmap to clinical, translational research (2021) [20]	33 publications—12 (human plasma), 9 (14 different matrices in humans) and 12 (21 different species/matrices).	14–103 years	LC-MS, SOMAscan, PEA	232
21	Systematic review and analysis of human proteomics aging studies unveils a novel proteomic aging clock and identifies key processes that change with age (2020) [69]	11,225 people from 32 studies	<1–95 years	LC-MS/MS, SOMAscan, 2D gel electrophoresis, SWATH-MS.	1128/32
22	Meta-analysis of age-related gene expression profiles identifies common signatures of aging (2009) [12]	27 datasets (12 mouse experiments, 11 rat experiments, and 4 human experiments)	20–106 years	Microarrays	56

## Data Availability

The raw data supporting the conclusions of this article will be made available by the authors on request.

## Supplementary Materials

The following supporting information can be downloaded at https://www.mdpi.com/article/10.3390/proteomes13040057/s1, Figure S1_venn_pairwise_databases; Figure S2_venn_pairwise_researches; Table S1. Comparison of advantages and disadvantages of proteomics data acquisition methods; Table S2. Comparison of characteristics of proteomics data acquisition methods; Table S3. Characteristics of major proteomic databases; Table S4. Analysis of publication selection criteria for proteomic databases; Table S5. Comparative table of designs and characteristics of proteomic studies; Table S6. Comparative table of designs and characteristics of proteomic studies; File S1. upset_analysis_results_DB.xlsx; File S2. upset_analysis_results_Research.xlsx.
