# Supplementary material for: Integrated Analysis of Proteomic Marker Databases and Studies Associated with Aging Processes and Age-Dependent Conditions: Optimization Proposals for Biomedical Research
Source: Proteomes. 2025 Nov 6;13(4):57. doi: 10.3390/proteomes13040057 (PMC12641871; doi:10.3390/proteomes13040057)
Supplement: Supplementary file 1 [file proteomes-13-00057-s001.zip › Tables_eng.pdf]

Table S1. Comparison of advantages and disadvantages of proteomics data acquisition methods

Method	Principle	Advantages	Disadvantages
**Protein Pathway Array** (PPA)	Multiplexed immunoassay on chip for pathway analysis	High-throughput analysis of protein regulatory networks	Limited to known antibody sets, does not detect novel proteins
**Tissue Microarray** (TMA)	IHC on hundreds of samples simultaneously	Mass verification of tissue biomarkers; protein localization determination	Depends on antibody quality; typically no absolute concentration quantification; quite expensive and labor-intensive
**Luminex** (Bead Array)	Multiplexed sandwich ELISA with fluorophore-labeled beads	Rapid analysis of dozens to hundreds of proteins simultaneously	Lower sensitivity than specialized platforms; inter-platform differences
**MSD** (Meso Scale Discovery)	Multiplexed analysis on electrochemiluminescent platform	Very sensitive for cytokines; high linearity	Complex calibration; sometimes lower specificity than Luminex
**Simoa** (Quanterix)	Digital multiplexed ELISA	Detects proteins at fg/ml levels	Limited to small number of simultaneous targets (6-10); high cost of assays
**SOMAscan** (SomaLogic)	Multiplexed analysis with protein aptamers	Analysis of up to 11,000 proteins; sensitivity up to 40 fM; wide dynamic range	Requires complex bioinformatics; sometimes discrepancies with other methods are observed
**Olink** (PEA)	PEA – each antibody pair is linked to DNA probes; upon label capture, a unique DNA fragment is synthesized	Analysis of thousands of proteins (panels up to 5000 targets) from microscopic volumes; high sensitivity/specificity	High-quality antibodies required; qPCR analysis requires separate instrument
**Alamar NULISA** (PEA+)	Advanced PEA with dual antibody capture and release	Attomolar sensitivity; authors claim improved dynamics and reliability compared to Olink	New technology, limited availability; independent evaluations still limited

**Oxford Nanopore**	Reading amino acid sequence of protein through nanosensor	Potential for sequencing single protein molecules without labels	Low efficiency, high reading error rate

Table S2. Comparison of characteristics of proteomics data acquisition methods

Method	Operating principle	Throughput	Measurement accuracy	Analysis cost	Analysis time	Main application in aging	Limitations
**Protein Pathway Array** (PPA)	Antibodies on solid surface	Medium (50-300 proteins)	90-98% specificity	$100-300/sample	2-4 hours	Inflammatory aging markers	Cross-reactions
**Tissue Microarray** (TMA)	Next-generation tissue microarrays	Low (10-100 proteins)	85-95% specificity	$300-800/sample	1-2 days	Age-related tissue pathology	Low throughput
**Luminex** (Bead Array)	Multiplex fluorescent beads	Medium (25-500 proteins)	95-99% specificity	$150-400/sample	4-8 hours	Aging cytokines and hormones	Limited multiplexing
**Simoa** (Quanterix)	Digital ELISA detection	Very low (1-20 proteins)	99-99.9% specificity	$50-200/marker	2-6 hours	Ultra-sensitive biomarkers	High cost per marker
**SOMAscan** (SomaLogic)	DNA aptamers (proteomic analysis)	Very high (7000+ proteins)	90-98% specificity	$1500-3000/sample	1-2 days	Systems biology of aging	Very high cost
**DIA-MS**	Data-Independent Acquisition	High (2000-8000 proteins)	±1-3 ppm	$300-700/sample	3-8 hours	Quantitative aging proteomics	Data processing complexity

Table S3. Characteristics of major proteomic databases

№	Database	Data volume (2024)	API access	Download formats
**1**	AgeAnnoMO	Samples 8586, Dataset 136	No (Github or Zenodo)	XLSX
**2**	AgeFactDB	Ageing Factors 16599<br>Genes 16450<br>Compounds 91<br>Other Ageing Factors 58	No	TSV, CSV and XML
**3**	Aging Atlas	1133 Aging factors	No	JSON, CSV, TSV
**4**	AgingBank	503 genes across various Hallmarks of aging	No	TXT
**5**	HAGR	Human genes: 307	No	CSV, TXT
**6**	HALL	500+ researchs	No	JSON, CSV
**7**	Human Protein Atlas	Consists of 1162 proteins quantified by Proximity Extension Assay (PEA) and 146 proteins quantified by isotope dilution strategies	No	CSV, TXT
**8**	iProX	2,521 datasets for humans	No	mzML, mzTab
**9**	jMorp	Results of proteome analysis of approximately 500 Japanese plasma samples	No	Images, metadata

**10**	jPOSTrepo	3573 projects are registered. 2668 are opened.<br>460 species.	No	mzML, pride XML
**11**	KEGG	Pathway maps 580<br>Human diseases 2,963	Yes (REST)	CSV, TXT
**12**	MassIVE	Public Datasets: 17,711, Proteins: 191,740, Number of Files: 11,158,091, Peptides: 9,906,636	No	mzML, mzXML, MGF
**13**	MetaboAge DB	1,500+ metabolites	Yes (REST)	SDF, CSV
**14**	PRIDE Archive	Contains over 42,000 datasets	Yes (REST)	mzML, mzTab, RAW
**15**	STRING	Homo sapiens has 19488 proteins with network connections	Yes (REST)	CSV, TXT
**16**	UniProt	220+ million records	Yes (REST)	FASTA, XML, TSV

Note: REST (Representational State Transfer); FTP (File Transfer Protocol); dbGaP (Database of Genotypes and Phenotypes)

Table S4. Analysis of publication selection criteria for proteomic databases

**№**	**Database**	**Methodological requirements**	**Journal quality (IF)**	**Sample size**	**Demographic criteria**	**Ethical requirements**
**1**	AgeAnnoMO (Huang et al., 2024)	Genetic experiments	IF ≥2.0	≥3 biol. replicates	Model organisms only	Standart

**2**	AgeFactDB<br>(Hühne et al., 2014)	Aging factors	IF ≥4.0	Meta-analyses	Different populations	Standart
**3**	Aging Atlas<br>(Hou et al., 2020)	Aging omics data	IF ≥3.0	≥20 samples/group	Age >40 years	IRB approval
**4**	AgingBank<br>(Gao et al., 2020)	Aging genes/proteins	IF ≥3.0	≥20 samples	Model organisms + human	Standart
**5**	HAGR<br>(Tacutu et al., 2018)	Longevity/gerontology	IF ≥4.0	Large cohorts	Human, centenarians	Strict ethical requirements
**6**	HALL<br>(Li et al., 2024)	Healthy aging	IF ≥3.0	≥50 healthy individuals	Age 65+ healthy	Strict IRB
**7**	Human Protein Atlas	Immunohistochemistry, transcriptomics	High-impact journals	Tissue-specific samples	Human tissues/cells	Swedish ethical approval
**8**	iProX<br>(Ma et al., 2019)	Proteomic MS data	Chinese/English journals	≥5 biol. replicates	Priority to Asian populations	Chinese standards
**9**	jMorp<br>(Tadaka et al., 2023)	Morphological data	IF ≥2.0	≥10 images/group	Japanese population	Imaging consent
**10**	jPOSTrepo<br>(Okuda et al., 2017)	Proteomic data	Japanese journals IF ≥1.0	≥5 samples	Priority to Japanese cohorts	Japanese standarts

**11**	KEGG	Pathway mapping	Peer-reviewed	Not specified	Many organisms	Standard
**12**	MassIVE (Wang et al., 2018)	Any MS data	No restriction	No restriction	No restriction	Basic
**13**	MetaboAge (Bucaciuc Mracica et al., 2020)	Aging metabolomics	IF ≥2.5	≥30 samples	Age specification required	Standart
**14**	PRIDE (Perez-Riverol et al., 2025)	MS/MS data, standard formats	IF ≥1.0	≥10 samples	Species specification required	Ethics committees
**15**	STRING	Protein interactions	High-quality sources	Literature-based	All organisms	Standard
**16**	UniProt (Apweiler et al., 2004; Choudhary et al., 2013)	Experimental validation	Any peer-review	Not specified	All species/populations	Standart

Table S5. Comparative table of designs and characteristics of proteomic studies

№	Abbreviation	Samples	Age distribution	Study type	Reference
1	BLSA, GESTALT	240	22-93	Cross-sectional	Tanaka et al., 2018
2	LonGenity, INTERVAL	4263	18-95	Longitudinal	Lehailler et al., 2019
3	LonGenity	1025	65-95	Longitudinal	Sathyan et al., 2020
4	DESIGN,20157ATSLF	86	-	Cross-sectional	Siino et al., 2022
5	CN-Beijing-2012	1890	18-82	Cross-sectional	Lu et al., 2012

6	CN-Bama-2019	66	-	Cross-sectional	Ye et al., 2019
7	IT-2020	18	67-81,100±3	Cross-sectional	Santos-Lozano et al., 2020
8	CN-2020	118	21->60	Cross-sectional	Xu et al., 2020
9	PIVUS	2016	70-80	Longitudinal	Lind et al., 2019
10	InCHIANTI study	997	21-102	Cross-sectional	Tanaka et al., 2020
11	CN-Bama-2018	66	32-56	Cross-sectional	Wang et al., 2018
12	ABF300, HERITAGE, LonGenity, deCODE	37650	16-105	Cross-sectional	Coenen et al., 2023
13	CHS, FOS	3984	76.3±5.0,54.2±9.5	Longitudinal	Liu et al., 2022
14	LonGenity, INTERVAL	5676	64-95	Cross-sectional	Oh et al., 2023

Meta-analysis

№	Abbreviation	Description	Reference
1	Age prediction from human blood plasma using proteomic and small RNA data	In this study, protein and small RNA levels were examined in 103 human blood plasma samples. Initially, a two-step mass spectrometry approach was used to measure 612 proteins in order to select and quantify 21 proteins that changed in abundance with age	Salignon et al., 2023
2	Global analysis of aging-related protein structural changes uncovers enzyme-polymerization-based control of longevity	Comparative analysis of protein structural states between young and old cells revealed age-dependent structural differences in 468 proteins, which included 1,272 conformation-specific peptides (total proteins detected: 2,833	Jurgita Paukštytė et al., 2023
3	Markers of aging	5,000 proteins in humans (37,479 individuals) aged 16 to 95 years across all cohorts from four independent large-scale studies. (150, 25-80; 745, 16-66; 1025, 65-95; 35559, mean 55	Coenen et al., 2023
4	Organ aging signatures in the plasma proteome track health and disease	4,979 proteins in 5,676 individuals across five independent cohorts (Covance n=1029, 19-89; LonGenity n=962, 61-95; SAMS, n=192, 60-88; Stanford-ADRC, n=409, 36-93; Knight-ADRC AD, n=1677, 27-101)	Oh et al., 2023
5	Proteomic aging clock predicts mortality and risk of common age-related diseases in diverse populations	UK Biobank (45,441, 39–71 years); China Kadoorie Biobank (3,977 CKB, 30–78 years); FinnGen (1,990 Finnish, 19–78 years)	Argentieri et al., 2024
6	Proteomics in aging research: A roadmap to clinical, translational research	33 publications – 12 (human plasma), 9 (14 different matrices in humans) and 12 (21 different species/matrices). Age range from 14 to 103 years	Moaddel et al., 2021

7	Systematic review and analysis of human proteomics aging studies unveils a novel proteomic aging clock and identifies key processes that change with age	32 publications, age range from neonatal (<1 year) to 95 years. Total number of participants for 32 publications: 11,225	Johnson et al., 2020
8	Meta-analysis of age-related gene expression profiles identifies common signatures of aging	27 datasets (12 experiments with mice, 11 with rats and 4 with humans)	Magalhães et al., 2009

Table S6. Comparative table of designs and characteristics of proteomic studies

№	Gene protein	Description	Function	Frequency of occurrence in sources
1	PTN	Pleiotrophin	Involved in tissue regeneration and neuroplasticity	5 times
2	ALB	Albumin	Level changes associated with inflammation and aging	5 times
3	GDF15	Growth differentiation factor 15	Biomarker of stress and aging, affects metabolism	4 times
4	SOST	Sclerostin	Regulates bone remodeling and bone density	3 times
5	MXRA8	Matrix-regulating protein 8	Involved in inflammatory processes and bone tissue metabolism	3 times
6	KLK3	Kallikrein 3 (Prostate-specific antigen, PSA)	Associated with inflammation and prostate disease risk	3 times
7	CTSF	Cathepsin F	Participant in lysosomal pathway, important for cellular renewal	3 times
8	MMP12	Matrix metalloproteinase 12	Extracellular matrix degradation, associated with connective tissue aging	3 times
9	LYZ	Lysozyme	Immune function, influence on inflammatory processes	3 times
10	DSG2	Desmoglein 2	Important for cell adhesion and tissue regulation	3 times
11	ITGB3	Integrin beta-3	Participation in cell adhesion and signaling	3 times
12	SERPING1	Serpin P1	Regulation of complement system, associated with inflammation	3 times
13	VWF	Von Willebrand factor	Participates in coagulation and vascular dysfunction	3 times
14	VEGFA	Vascular endothelial growth factor A	Regulation of angiogenesis, important for vascular health	3 times
15	ADAMTS5	ADAMTS5	Proteoglycan breakdown, affects joint health	2 times

16	SCARF2	Clusterin and HDL receptor family protein 2	Affects cellular homeostasis and aging	2 times
17	WFDC2	WAP twelve-repeat protein domain	Associated with modulation of inflammatory responses	2 times
18	CGA	Corticotropin alpha-subunit (glycoprotein gamma)	Associated with endocrine functions and aging	2 times
19	RSPO4	R-spondin 4	Regulation of Wnt signaling pathway, important for cell proliferation	2 times
20	PTGDS	Prostaglandin D2 synthase	Participation in inflammatory processes and tissue protection	2 times
21	DPT	Dermatopontin	Associated with extracellular matrix functions and age-related skin changes	2 times
22	WISP-2	Dickkopf-related protein 2	Participates in cellular processes and tissue repair	2 times
23	FBLN1	Fibulin 1	Regulates structural changes in tissue	2 times
24	FGA	Fibrinogen alpha chain	Associated with coagulation and inflammatory processes	2 times
25	TPM1	Tropomyosin 1	Important for cellular structure, affects cardiac functions	2 times
26	GBA	Glucocerebrosidase	Lysosomal activity, affects cellular condition	2 times
27	B2M	Beta-2-microglobulin	Inflammation biomarker, its level increases with age	2 times
28	C1S	Complement component C1S	Participation in immune processes and inflammation	2 times
29	C9	Complement component C9	Part of complement system, affects inflammatory processes	2 times
30	CD14	Cluster of differentiation 14	Marker of inflammatory reactions and macrophage activation	2 times
31	CRTAC1	Cartilage oligomeric matrix protein 1	Associated with extracellular matrix functions	2 times
32	EFEMP1	Agrin-like 1	Participates in matrix remodeling and maintaining tissue structure	2 times
33	IGFALS	Insulin-like growth factor binding protein	Associated with metabolism regulation and aging	2 times
34	TGFBI	Transforming growth factor beta-induced	Participant in fibrosis and inflammation processes	2 times
35	APOC1	Apolipoprotein C1	Affects lipid metabolism and inflammation	2 times
36	SMOC1	SPARC-related modular calcium binding 1	Participates in angiogenesis regulation	2 times
37	CCL21	C-C motif chemokine ligand 21	Associated with immune cell migration and inflammation	2 times
38	IGFBP3	Insulin-like growth factor binding protein 3	Regulates cell growth and apoptosis	2 times
39	EGFR	Epidermal growth factor receptor	Regulation of cell growth and aging	2 times
40	AGRP	Agouti-related protein	Influence on metabolism and energy balance	2 times
41	CDH5	Cadherin 5	Important for cell adhesion and vascular function	2 times

42	FSHB	Follicle-stimulating hormone beta subunit	Regulation of reproductive processes	2 times
43	RET	RET receptor tyrosine kinase	Associated with nervous tissue and development	2 times
44	CTSV	Cathepsin V	Participates in proteolysis, influence on inflammation	2 times
45	KLK7	Kallikrein 7	Protein degradation, affects skin aging processes	2 times
46	FSTL3	Follistatin-like 3	Regulation of cell and tissue growth	2 times
47	CDON	Cell surface receptor CDON	Cell interaction, involved in tissue development	2 times
48	CLU	Clusterin	Important for apoptosis and protection from oxidative stress	2 times
49	SELL	L-selectin	Affects leukocyte adhesion and inflammatory reactions	2 times
50	FAS	Fas apoptotic antagonist	Participates in apoptosis, important for immune processes	2 times
51	CTSD	Cathepsin D	Lysosomal activity, important for cellular renewal	2 times
52	TFF2	Trefoil factor 2	Affects mucosal protection and inflammation	2 times
53	TREM2	TREM2	Regulation of immune reactions in nervous tissue	2 times
54	CA3	Carbonic anhydrase 3	Participates in pH balance and metabolism	2 times
55	BGN	Biglycan	Important for tissue structure and matrix health	2 times
56	WARS1	Tryptophanyl-tRNA synthetase 1	Participation in protein synthesis and stress adaptation	2 times
57	APP	Amyloid precursor protein	Associated with neurodegeneration and aging diseases	2 times
58	NEFL	Neurofilament light	Important for nervous tissue structure and neuron health	2 times
59	NDRG1	DNA damage-inducible transcript 4	Participates in DNA repair and stress protection	2 times
60	MAPT	Microtubule-associated protein tau	Microtubule regulation, associated with neurodegeneration	2 times
61	GFAP	Glial fibrillary acidic protein	Astrocyte marker, important for neuroinflammation	2 times
62	VEGFA	Aspartate aminotransferase 1	Participates in metabolic processes and amino acid exchange	2 times
63	GOT1	Galectin-3	Modulation of cellular stress and immune responses	2 times
64	LGALS3	Tumor necrosis factor receptor 1A	Associated with inflammatory processes and cell death	2 times
65	TNFRSF1A	Plasminogen activator urokinase receptor	Influence on fibrinolysis and cell migration	2 times
66	PLAUR	Complement component C3	Complement system component, involved in inflammation	2 times
67	C3	Complement component C4A	Participation in immune reactions, important for complement system	2 times
68	C4A	Cathepsin S	Involved in proteolysis and immune processes	2 times
69	CTSS	Pleiotrophin	Involved in tissue regeneration and neuroplasticity	5 times
