# Supplementary figures and images for: Integrated Analysis of Proteomic Marker Databases and Studies Associated with Aging Processes and Age-Dependent Conditions: Optimization Proposals for Biomedical Research
Source: Proteomes. 2025 Nov 6;13(4):57. doi: 10.3390/proteomes13040057 (PMC12641871; doi:10.3390/proteomes13040057)

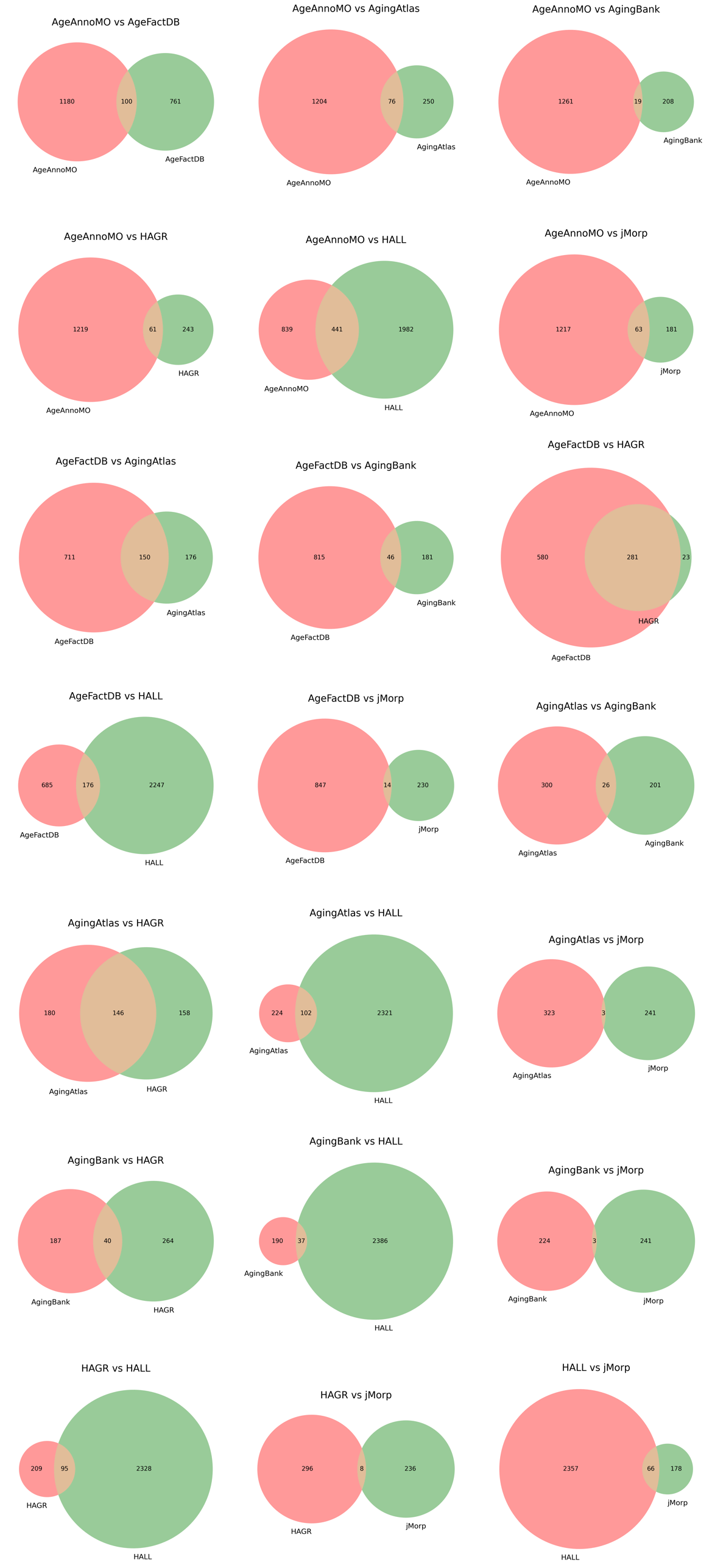

Supplement: Supplementary file 1 [file proteomes-13-00057-s001.zip › Figure S1_venn_pairwise_databases.png]
